# Identification of Gene Networks for Residual Feed Intake in Angus Cattle Using Genomic Prediction and RNA-seq

**DOI:** 10.1371/journal.pone.0152274

**Published:** 2016-03-28

**Authors:** Kristina L. Weber, Bryan T. Welly, Alison L. Van Eenennaam, Amy E. Young, Laercio R. Porto-Neto, Antonio Reverter, Gonzalo Rincon

**Affiliations:** 1 VMRD Genetics R&D, Zoetis Inc., Kalamazoo, MI, United States of America; 2 Department of Animal Science, University of California Davis, Davis, CA, United States of America; 3 CSIRO Agriculture, Queensland Bioscience Precinct, St. Lucia, QLD, Australia; University of Lleida, SPAIN

## Abstract

Improvement in feed conversion efficiency can improve the sustainability of beef cattle production, but genomic selection for feed efficiency affects many underlying molecular networks and physiological traits. This study describes the differences between steer progeny of two influential Angus bulls with divergent genomic predictions for residual feed intake (RFI). Eight steer progeny of each sire were phenotyped for growth and feed intake from 8 mo. of age (average BW 254 kg, with a mean difference between sire groups of 4.8 kg) until slaughter at 14–16 mo. of age (average BW 534 kg, sire group difference of 28.8 kg). Terminal samples from pituitary gland, skeletal muscle, liver, adipose, and duodenum were collected from each steer for transcriptome sequencing. Gene expression networks were derived using partial correlation and information theory (PCIT), including differentially expressed (DE) genes, tissue specific (TS) genes, transcription factors (TF), and genes associated with RFI from a genome-wide association study (GWAS). Relative to progeny of the high RFI sire, progeny of the low RFI sire had -0.56 kg/d finishing period RFI (P = 0.05), -1.08 finishing period feed conversion ratio (P = 0.01), +3.3 kg^0.75 finishing period metabolic mid-weight (MMW; P = 0.04), +28.8 kg final body weight (P = 0.01), -12.9 feed bunk visits per day (P = 0.02) with +0.60 min/visit duration (P = 0.01), and +0.0045 carcass specific gravity (weight in air/weight in air—weight in water, a predictor of carcass fat content; P = 0.03). RNA-seq identified 633 DE genes between sire groups among 17,016 expressed genes. PCIT analysis identified >115,000 significant co-expression correlations between genes and 25 TF hubs, i.e. controllers of clusters of DE, TS, and GWAS SNP genes. Pathway analysis suggests low RFI bull progeny possess heightened gut inflammation and reduced fat deposition. This multi-omics analysis shows how differences in RFI genomic breeding values can impact other traits and gene co-expression networks.

## Introduction

The largest variable costs in beef cattle production are feed and land [1]. Feed costs can be reduced by improving feed conversion efficiency without sacrificing production traits; one option for this is to consider residual feed intake (RFI; [2]). RFI is the difference between observed feed intake and expected intake based on body size and rate of production (growth, milk production, etc.) over a period of time. An animal with a low RFI has improved feed conversion efficiency since it consumes less feed than expected for its maintenance and growth requirements. The heritability of RFI has been estimated to be as high as 42% in growing beef cattle [3], suggesting it has a strong genetic component and would respond to selective breeding. While it is too expensive for all bull testing centers and feedlots to measure individual feed intake in order to calculate RFI, reference populations of animals possessing both RFI phenotypes and high density SNP genotypes have been used to generate predictions of total genetic merit [4] for RFI in major cattle breeds [5–7]. These genomic predictions can be a selection tool in the absence of direct phenotypic data.

The aim of this study was to determine whether progeny of bulls with divergent genomic predictions for RFI displayed physiological and transcriptomic differences. We used two influential Angus bulls with high or low genomically predicted breeding values for RFI to produce steer progeny, which were phenotyped for RFI and a large variety of traits from post-weaning through slaughter and carcass evaluation. Key tissues for growth and metabolism, including the pituitary, skeletal muscle, liver, visceral adipose, and duodenum, were collected for RNA-seq analysis. Partial correlation and information theory (PCIT) was used to generate gene networks, linking tissue specific (TS) and differentially expressed (DE) genes with known transcription factors (TF) and genes harboring SNP from a large genome-wide association study (GWAS) study. The DE genes and gene networks described here provide new insights into regulatory and gene expression pathways of RFI.

## Materials & Methods

### Animal Management and Phenotype Collection

Using genomic predictions for RFI in Angus cattle [8] produced by Zoetis Inc. (Florham Park, NJ), two influential purebred Angus bulls were selected as breeding sires. One bull was predicted to be in the top 1% in the Angus breed in 2010 for RFI, and the other in the bottom 10%, with a difference in RFI breeding value of 0.32 kg DM/d or approximately 3.5% of daily intake. Semen from these bulls was used to inseminate a group of commercial cows of predominantly Angus background at the University of California Sierra Foothill Research and Extension Center (Browns Valley, CA). Eight steer offspring per sire were selected for participation in the study, matched between sire groups by weaning weight. Eight animals per group provides 80% power to detect differential gene expression with fold change greater than or equal to 1.8 for the top 80% of genes by coverage using a biological coefficient of variation of 0.4 and alpha of 0.05 ([9]). The use of half-sibling progeny of two bulls is expected to reduce the variability within low or high RFI populations, but does confound RFI with other traits for which the bulls differ. Sire differences for other breeding values are provided in S1 Table. Based on molecular and traditional breeding values, the low RFI sire is predicted to have lower intake, higher weights from birth through to carcass, larger height and rib-eye area, and reduced backfat thickness.

All livestock were managed under protocols approved by the Institutional Animal Care and Use Committee (IACUC) of the University of California. Post weaning, at ~ 8 mo of age, the animals were transferred to a commercial feedlot (Snyder Livestock Co., Yerington, NV; http://www.slcnv.com/), where they were housed in a single pen equipped with GrowSafe units (GrowSafe System Ltd., Airdrie, AB, Canada) for daily feed intake measurement over a period of 70 d (henceforth referred to as the growing period) after a 14 d adaptation period. Animals were fed a standard grower diet (Table 1). Feed was provided daily in the morning and pushed towards the cattle two additional times during the day to encourage feeding. Feed intake was measured continuously to the 0.01 kg by the GrowSafe system. Data from 2 d were omitted due to system failure. Feeding behavior was assessed based on frequency and duration of bunk visits. Body weights were taken every 14 d.

**Table 1:** Composition of the total mixed ration (TMR) used in the growing and finishing periods. Finishing diet possessed a higher proportion of grain and higher energy content (Mcal/kg).

Item	Growing	Finishing,Mean (SE)
Ingredient, %		
Corn silage	32.56	
Corn steepwater	2.75	
Rolled corn		62.65
Distillers grain		17.23
Almond hulls	20.00	
Rice bran	18.28	
Wheat	10.00	
Alfalfa hay	8.00	7.83
Oat hay	2.00	3.91
Molasses		4.74
Condensed molasses solubles	2.89	
Whey	2.30	
Fat		1.96
Limestone		1.28
Calcium carbonate	0.50	
White salt	0.46	0.26
Suspension agent	0.18	
Magnesium oxide		0.13
Trace minerals	0.06	
Rumensin	0.02	0.01
Analyzed, %DM basis		
Crude protein	11.36	13.30 (0.86)
Crude fiber	16.35	
ADF		9.00 (1.43)
NDF		16.80 (1.34)
Calculated		
NEm [10], Mcal/kg	1.263	2.112 (0.047)
NEg [10], Mcal/kg	0.723	1.437 (0.039)

Following the growing period, the animals were transferred to the University of California, Davis Animal Science Feedlot facility, where they were individually housed in 7.5 m^2^ (1.5 x 5 m) pens for a minimum of 70 d after a 14 d adaptation period or until they reached a body condition appropriate for slaughter (91 d on average), hereafter referred to as the finishing period. Total feed offered and amount refused were measured to 0.05 kg precision, and daily intake was calculated as the difference between offered and refused feed per animal per day. Body weights were measured every 14 d before AM feeding.

For five randomly selected days during the finishing period, the daily ration was provided via a tie-stall GreenFeed unit (C-Lock Inc., Rapid City, SD) in order to collect methane emission measurements during feeding. For one of those days, the timing and quantity of feed consumed from the GreenFeed unit was controlled to minimize variation due to animal feeding behavior. During that day, approximately 1 kg of total mixed ration (TMR) was provided at alternating 1.5 and 3 h intervals in order to measure the peaks and troughs of methane production [11]; on the other days, feed provided from the GreenFeed unit was a pelleted form of the finishing period TMR components, provided ad libitum. For non-GreenFeed days, daily ration was divided into four parts offered throughout the day (early and late morning and afternoon) in order for the intermittent feeding allowed by the GreenFeed to not present a huge departure from habituated feeding patterns.

For both the growing and finishing periods, RFI is defined as the residual of dry matter intake adjusted for rate of gain and body size [12],

$$\mathrm{DMI}=b_{0}+b_{1}\mathrm{ADG}+b_{2}\mathrm{MMW}\;+\mathrm{RFI}$$

where DMI is average daily dry matter intake; ADG is average daily gain, estimated from the regression of 14d-body weights on time; MMW is metabolic mid-weight, or (mean period body weight)^0.75^; **b**_**0**_, **b**_**1**_ and **b**_**2**_ are the partial regression coefficients associated with the intercept, ADG and MMWT, respectively.

At slaughter, standard beef carcass trait phenotypes were collected, with the addition of organ weights and carcass specific gravity, a predictor of the fat content of the carcass [13], estimated from CW/(CW-CW_H2O_) where CW is the dry carcass weight and CW_H2O_ is the weight of the carcass suspended in water (measured to nearest 1g).

### RNA Isolation, cDNA Library Construction, and Transcriptome Sequencing

Samples of pituitary, skeletal muscle, liver, visceral adipose (KPH fat), and duodenum were collected within 25 minutes of slaughter, snap frozen in liquid nitrogen to preserve RNA integrity, and frozen at -80°C. To collect the pituitary, the skull was bisected longitudinally and the pituitary identified by a bovine physiologist. To extract RNA, approximately 200 mg of frozen tissue was immersed in liquid nitrogen, ground with a mortar and pestle, and homogenized in 2 mL TRIzol® (Thermo Fisher Scientific, Waltham, MA) using a Mini BeadBeater-8 (Biospec Products, Bartlesville, OK) for up to 10 s, except fat tissue which was shaken rather than bead beaten to avoid RNA degradation. Total RNA was purified using the TRIzol standard protocol (Thermo Fisher Scientific, Waltham, MA), and resuspended in 25μL of RNase-free water. RNA quantity and quality were assessed using NanoDrop® spectrophotometer (Thermo Scientific, Wilmington, DE) and Agilent 2100 Bioanalyzer (Santa Clara, CA). For each tissue type, mean 260/280 ratio ranged from 1.84 to 1.93 (0.10 SD), and mean RNA integrity number from 7.0 to 8.1 (2.0 SD). The Illumina TruSeq stranded mRNA HT sample preparation protocol (San Diego, CA) was used to generate dual-indexed cDNA libraries for each sample using 1 μg of total RNA as input. Success of library preparation was determined by Agilent 2100 Bioanalyzer (Santa Clara, CA), after which all 80 samples were multiplexed into a single equimolar pool to avoid sequencing run bias. Single end 100 bp sequencing was conducted on an Illumina HiSeq 2000 following standard protocols.

### Sequence Analysis and Mixed Model Analysis

Eighty samples, five tissues from sixteen Angus steers, were analyzed with RNA-seq. Failed reads were discarded before analysis. Samples with less than 10 million reads (one adipose and one duodenal sample) were discarded. For the remaining samples, we obtained 45.2 M reads per sample on average, with mean Phred score 35.8 (0.13 SD), median Phred 38, and less than 7% of reads below Phred score 30. No additional sequence filtering was performed. Sequence reads were mapped to the bovine reference genome (UMD3.1 for BTA1-29 and X and Btau 4.6.1 for BTAY) using CLC Genomics Workbench 7 RNA-seq tool (Redwood City, CA). Reads were required to map with 80% similarity for 90% of their length, which 93% of reads achieved. We used reads per kilobase of gene per million mapped reads, RPKM [14], as the unit of expression. A gene was considered tissue specific (TS) if one tissue represented at least two-thirds of the total expression across tissues. Highly expressed TS genes were compared with tissue expression profiles in *Bos taurus* and other vertebrate species based on the EMBL-EBI Expression Atlas (https://www.ebi.ac.uk/gxa/home) to verify that they were consistent with previously observed expression patterns in these tissues.

Gene expression by sire group was estimated using a mixed model approach [15, 16] in order to determine whether any genes were differentially expressed across tissues due to sire effects.

$$Y_{\mathrm{ijkl}}=\mu +G_{i}+GT_{\mathrm{ij}}+GA_{\mathrm{ik}}+GS_{\mathrm{il}}+e_{\mathrm{ijkl}}$$

where log 2-transformed RPKM (Y_ijkl_) was modeled as a function of the random effects of gene (G_i_), gene by tissue (GT_ij_), gene by animal (GA_ik_), and gene by sire group (GS_il_) for *i* genes, *j* tissues, *k* animals, and *l* sires. Random residual (e_ijkl_) was assumed to be independent and identically distributed. Variance component analysis was performed using VCE6 software (Eildert Groeneveld, Friedrich-Loeffler-Institut, ftp://ftp.tzv.fal.de/pub/vce6/). A gene was considered differentially expressed between sire groups (DE) if the gene by sire effect was at least two standard deviations from the mean, corresponding to P<0.01. To compare TS and DE genes with known transcription factors (TF), all known TF from AnimalTFDB [17] (http://www.bioguo.org/AnimalTFDB/) were filtered for those both abundant and most consistently associated with TS and DE genes between sire groups based on regulatory impact factor metrics [16, 18] which weights average gene expression across samples (G_i_), the differential expression due to sire group (GS_il_), and the co-expression correlation between TF and DE or TS genes. Genes harboring SNP identified by Bolormaa et al. [19] as associated with RFI (hereafter referred to as SNP genes) were included to determine how closely this experiment aligns with a GWAS study from a broader beef cattle population.

### Network and Pathway Analysis

A gene network for RFI within these five tissues was derived using the TS, DE, TF and SNP genes as nodes, and significant connections between them were determined via partial correlation and information theory (PCIT) algorithms and software as described by Reverter and Chan [20]. PCIT relies on calculating the correlation between a pair of genes after accounting for all other genes (thus, the partial correlation). Connections between gene nodes were accepted when the partial correlation was greater than two standard deviations from the mean (P < 0.01). Pairwise co-expressed genes were imported into Cytoscape software [21] (http://www.cytoscape.org/), to be visualized and clustered into networks using a force-directed edge-weighted spring embedded layout [22]. Further functional analysis was performed using Ingenuity Pathway Analysis ® (Redwood City, CA; www.qiagen.com/ingenuity) and Blast2GO PRO [23]. For IPA analyses, the human/mouse/rat gene orthologue IDs and the fold changes for all DE bovine genes expressed in each tissue were compared to the IPA database of pathways, networks, diseases, functions, and regulators for gene set enrichment (P < 0.05) and activation Z-score (|Z| > 2) analysis. For Blast2GO, all annotated bovine genes were imported from Ensembl Biomart (http://www.ensembl.org/biomart/martview/), and significantly enriched GO terms (P<0.05) for a given gene subset were identified using Fisher’s exact test.

## Results

### Sire Genomic Breeding Values Predicted Difference in Progeny Phenotypes

The high and low RFI bulls’ progeny were phenotyped for RFI and a variety of growth, intake, metabolism, and body composition traits to determine which traits differentiated them. As predicted from their sires’ breeding values, the progeny of the low RFI sire had lower RFI than the progeny of the high RFI sire in the finishing phase (-0.57 kg DM/d), with a similar trend in the growing phase (-0.62 kg DM/d; Table 2). As with RFI, feed conversion ratio (DMI/ADG) was decreased in progeny of the low RFI sire relative to the high RFI sire in the finishing phase. Sire breeding values predict progeny of the low RFI sire to exhibit reduced intake with similar ADG relative to progeny of the high RFI sire, which was observed in the growing phase (sire group differences of -0.48 kg/d DMI and 0.00 kg/d ADG) but not in the finishing phase (sire group differences of 0.01 kg/d DMI and 0.22 kg/d ADG). Larger finishing and carcass weights were observed in the progeny of the low RFI sire, as might be predicted from the higher pedigree-based breeding values of that sire for weaning weight (WW), yearling weight (YW), and mature weight (MW). The low RFI sire group had higher carcass specific gravities, indicating that these animals were less fat or leaner for the same carcass weight. No significant differences were observed in other carcass traits or in heart, liver or kidney weights.

**Table 2:** Phenotypic differences between sire groups (low RFI sire–high RFI sire) compared with expected progeny difference based on bull breeding values. Differences between sire progeny groups for growth, intake, feed efficiency, feeding behavior, methane production, and carcass traits are provided, including trait, trait mean, mean difference between sire groups, P-value for that difference (P<0.05 bolded), and expected progeny difference based on sire breeding values where available.

Trait Type	Trait	Period	Mean (SE)	Sire Group Difference	P-value	Expected Progeny Difference
Growth,	MMW, kg^0.75^	Growing	74.5 (1.1)	0.8	7.3 E-01	
Intake, &		Finishing	100.4 (0.9)	3.3	**4.0 E-02**	
Feed	ADG, kg/d	Growing	1.71 (0.05)	0.00	9.8 E-01	0.00
Efficiency		Finishing	1.40 (0.06)	0.22	8.0 E-02	
	DMI, kg DM/d	Growing	9.55 (0.25)	-0.48	3.4 E-01	-0.18
		Finishing	8.52 (0.20)	0.01	9.8 E-01	
	Feed conversion	Growing	5.63 (0.17)	-0.28	4.1 E-01	
	ratio (DMI/ADG)	Finishing	6.21 (0.23)	-1.08	**1.0 E-02**	
	RFI, kg DM/d	Growing	0.00 (0.19)	-0.62	9.0 E-02	-0.16
		Finishing	0.00 (0.15)	-0.57	**5.0 E-02**	
Feeding	Bunk Visits, visits/d		57.7 (2.9)	-12.9	**2.0 E-02**	
Behavior	Bunk Visit Duration, min/visit		1.87 (0.13)	0.60	**1.0 E-02**	
(Growing Period)	Feeding Duration, min/d		96.7 (3.1)	7.04	2.6 E-01	
Methane Production	Controlled feed amount and interval, g CH_4_/d		165.3 (13.1)	6.6	8.1 E-01	
(Finishing Period)	Ad lib feeding, g CH_4_/d		161.8 (9.5)	8.7	6.6 E-01	
Carcass	Final weight, kg		533.9 (6.1)	28.8	**1.0 E-02**	MW 33.6
	Hot carcass weight, kg		323.4 (4.1)	12.6	1.1 E-01	10.5
	Rib-eye area, cm^2^		80.2 (1.9)	6.8	7.0 E-02	5.7
	Backfat thickness, mm		12.9 (0.4)	-0.5	5.4 E-01	-0.5
	Yield grade		3.0 (0.1)	-0.3	2.6 E-01	
	Marbling score		6.8 (0.3)	0.0	1.0 E+00	0.0
	Carcass specific gravity		1.08 (0.002)	0.0045	**3.0 E-02**	
	Heart weight, kg		2.01 (0.10)	0.25	2.0 E-01	
	Liver weight, kg		6.00 (0.17)	-0.03	9.2 E-01	
	Kidney weight, kg		1.00 (0.03)	-0.03	6.4 E-01	

While feeding behavior was only measured in the growing period, progeny of the low RFI sire visited the feed bunk less often (-12.9 visits/d) and stayed longer (0.6 min) at each visit. No significant differences were observed for methane production.

### Differential Expression and Pathway Analysis

**Fig 1:**
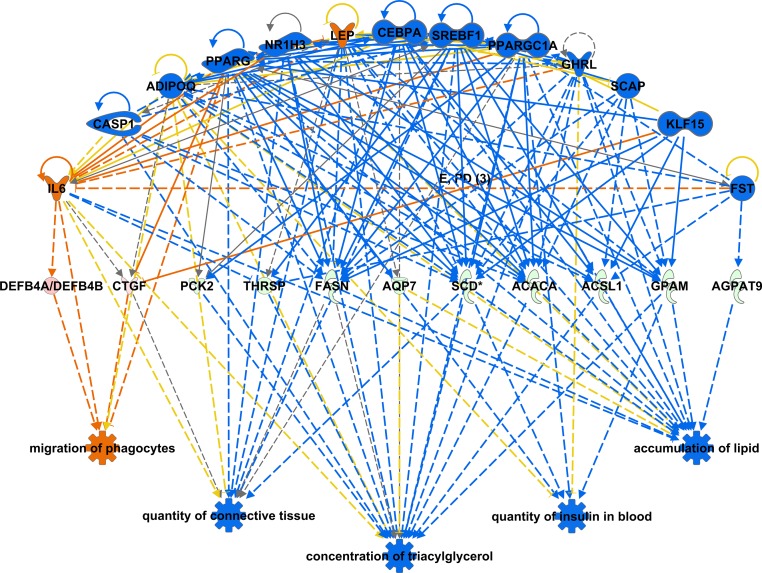
Downregulation of regulatory network controlling fat deposition among low RFI sire progeny. This figure displays the regulatory network connecting concentration of triacylglycerol, accumulation of lipid, quantity of insulin in the blood, quantity of connective tissue, and migration of phagocytes, overlaid with fold changes observed in adipose tissue. Red = up-regulated, green = down-regulated, orange = predicted activation, blue = predicted inhibition, and yellow = inconsistent.

Of 24,737 annotated bovine genes, 7,721 were not expressed (RPKM < 0.2) in any of the tissues surveyed. We identified 1,026 TS genes, including 285 in the pituitary, 220 in skeletal muscle, 275 in the liver, 33 in adipose, and 213 in the duodenum, and 633 DE genes across all tissues in the low versus high RFI sire groups (S2 Table). Pathway analysis showed most differentially expressed disease and biofunctions (P<0.05 and |Z|>2) corresponded to activation of the immune response in the duodenum and downregulation of fat deposition in adipose and muscle tissue (Table 3). The combined effect of downregulation of phosphoenolpyruvate carboxykinase 2 (*PCK2*), thyroid hormone responsive (*THRSP*), fatty acid synthase (*FASN*), stearoyl-CoA desaturase (*SCD*), acetyl-CoA carboxylase alpha (*ACACA*), acyl-CoA synthetase long-chain family member 1 (*ACSL1*), glycerol-3-phosphate acyltransferase (*GPAM*), and 1-acylglycerol-3-phosphate O-acyltransferase 9 (*AGPAT9*) support a deactivation of a regulatory network controlling fatty acid metabolism in the adipose tissue of the progeny of the low RFI sire (Fig 1). This is consistent with the increase in carcass specific gravity of the progeny of the low RFI sire, which suggests a reduced body fat content.

**Table 3:** Pathway analysis of differentially expressed genes between low and high RFI sire groups by tissue, for pituitary (P), skeletal muscle (M), liver (L), adipose (A), and duodenum (D). Top biofunctions by |Z-score| from IPA are provided, expressed as low relative to high RFI sire progeny group differences; all are significant (P<0.05). Functions associated with the immune system or fat deposition are grouped together, all other significant functions at the bottom.

Diseases or Functions Annotation	P	M	L	A	D	Diseases or Functions Annotation	P	M	L	A	D
Immune system						Fat deposition					
activation of antigen presenting cells					2.6	differentiation of adipocytes			2.4		
immune response of antigen presenting cells		2.0				mass of adipose tissue			2.2		
inflammation of body region		2.1				concentration of triacylglycerol		-2.5			
chemotaxis of granulocytes	2.0					synthesis of triacylglycerol	-2.4			-2.6	
recruitment of granulocytes					2.2	quantity of diacylglycerol					-2.0
inflammation of large intestine	-2.7					concentration of acylglycerol		-2.1			
colitis	-2.7					synthesis of acylglycerol				-2.2	
activation of leukocytes	2.3				2.7	metabolism of membrane lipid derivative			-2.2		
immune response of leukocytes		2.2				concentration of 1,2-dipalmitoylphosphatidylcholine	-2.3	-2.3		-3.1	
recruitment of leukocytes	2.7			2.8	2.8	concentration of phosphatidylcholine		-2.2		-2.1	
activation of macrophages					2.5	beta-oxidation of fatty acid		-2.2			
activation of mononuclear leukocytes	2.3					accumulation of lipid	-3.0			-2.4	
cell movement of mononuclear leukocytes					2.0	binding of lipid		2.0			
response of mononuclear leukocytes		2.6				flux of lipid	2.6		2.5		
activation of myeloid cells					2.3	transport of lipid	2.8				
response of myeloid cells		2.4				transport of cholesterol	2.1				
chemotaxis of neutrophils					2.3	fatty acid metabolism	2.1				
activation of phagocytes					2.4	transport of steroid	2.2				
immune response of phagocytes		2.4				steroid metabolism					-2.2
recruitment of phagocytes	2.5			2.1	2.4	concentration of hormone				-3.0	
response of phagocytes		3.0			2.2	binding of carbohydrate	2.0	2.2			
viral infection		-2.3				uptake of carbohydrate		2.1			
inflammatory response	2.3				3.2	insulin resistance					2.1
Other traits											
morbidity or mortality	-2.2					relaxation of artery			2.7		
necrosis	-2.5					recruitment of blood cells	2.4			2.6	2.6
deposition of extracellular matrix	-2.2				-2.2	vasoconstriction					2.0
contractility of muscle		2.2				bleeding					-2.3
proliferation of muscle cells		2.6				function of cardiac muscle		2.1			
formation of fibrils		-2.2				activation of cells	2.3			2.0	2.2
fibrogenesis			-2.0		2.2	cell movement	2.1				2.6
injury of liver					2.1	engulfment of cells					2.6
export of molecule	2.3					migration of cells	2.1				2.8
neurological signs				2.2		damage of endothelial cells		2.0	-2.2		
secretion of protein					2.2	damage of epithelial tissue		2.5			
soft tissue neoplasm					-2.1	stimulation of epithelial cells				-2.1	

**Fig 2:**
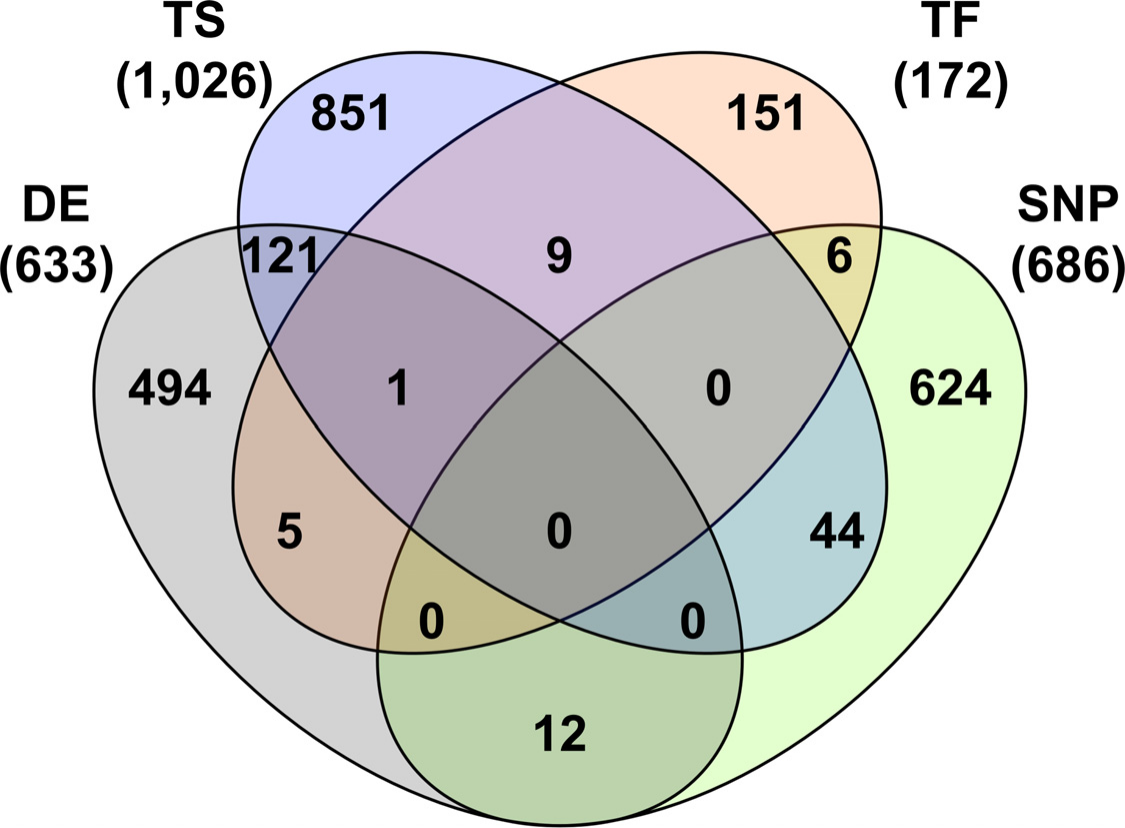
Venn diagram of DE, TS, TF, and SNP genes.

Of the 633 DE genes, 122 were TS, 6 were TF, and 12 harbor SNP identified by Bolormaa et al. [19] as associated with RFI in a large cattle population (Fig 2). The 6 DE TF are ETS variant 4 (*ETV4*), group-specific component/vitamin D binding protein (*GC*, also liver-specific), high mobility group box 1 protein (*HMGB1*), sex determining region Y-box 6 (*SOX6*), transducin beta-like 1 Y-linked (*TBL1Y*), and an uncharacterized protein (*ENSBTAG00000036343*). *ETV4* is most highly expressed in the pituitary and duodenum and is upregulated in the progeny of the low RFI sire. *HMGB1*, *SOX6*, and *TBL1Y* are expressed in all assayed tissues but most highly in the duodenum. *HMGB1* and *TBL1Y* are upregulated in all tissues, and *SOX6* is downregulated in the duodenum, pituitary, and skeletal muscle and upregulated in liver and adipose. *ENSBTAG00000036343* is an adipose-specific heat shock protein and strongly downregulated (-5.2 fold change). The 12 DE genes harboring GWAS SNP are acyl-CoA synthetase long-chain family member 1 (*ACSL1*), carcinoembryonic antigen-related cell adhesion molecule 20 (*CEACAM20*), chordin-like 2 (*CHRDL2*), claudin 2 (*CLDN2*), glypican 3 (*GPC3*), glutamate receptor ionotropic AMPA 2 (*GRIA2*), interleukin 2 receptor gamma (*IL2RG*), lipoma HMGIC fusion partner-like 1 (*LHFPL1*), lin-52 DREAM MuvB core complex component (*LIN52*), ryanodine receptor 3 (*RYR3*), transmembrane protein 8C (*TMEM8C*), and ZW10 interacting kinetochore protein (*ZWINT*). While relatively few DE genes are TF or SNP genes, some have key biological functions in the immune system, lipid metabolism, and muscle processes. *GC* is the primary carrier of vitamin D in the blood and a macrophage activating factor which is used in some cancer therapies [24]. *IL2RG* is a signaling component of many interleukin receptors and loss-of-function of this gene has been associated with X-linked immunodeficiency in humans [25]. *ACSL1* is an intermediate of fatty acid metabolism. *RYR3* is involved in releasing calcium from intracellular stores. Overall, many DE genes were TS and only a few were also TF or GWAS SNP genes, many supporting immune system and fat metabolism roles.

### PCIT Network Analysis

**Fig 3:**
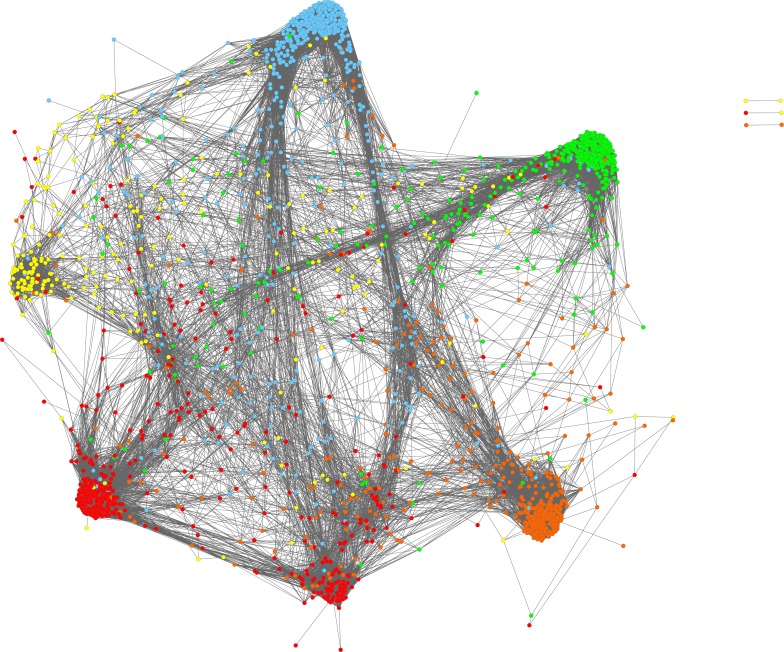
Gene co-expression network diagram including differentially expressed genes between sire groups, tissue specific genes, transcription factors regulating growth and feed efficiency, and genes harboring GWAS SNP for RFI. Genes (or nodes) are connected by an edge when a significant partial correlation was determined by PCIT. Colors represent the tissue of highest relative expression for each gene: pituitary (light blue), skeletal muscle (red), liver (light green), visceral adipose (yellow), and duodenum (orange).

Partial correlation information theory was used to determine significant partial correlations between TS, DE, TF, and SNP genes (N = 115,058 edges; Fig 3).

**Fig 4:**
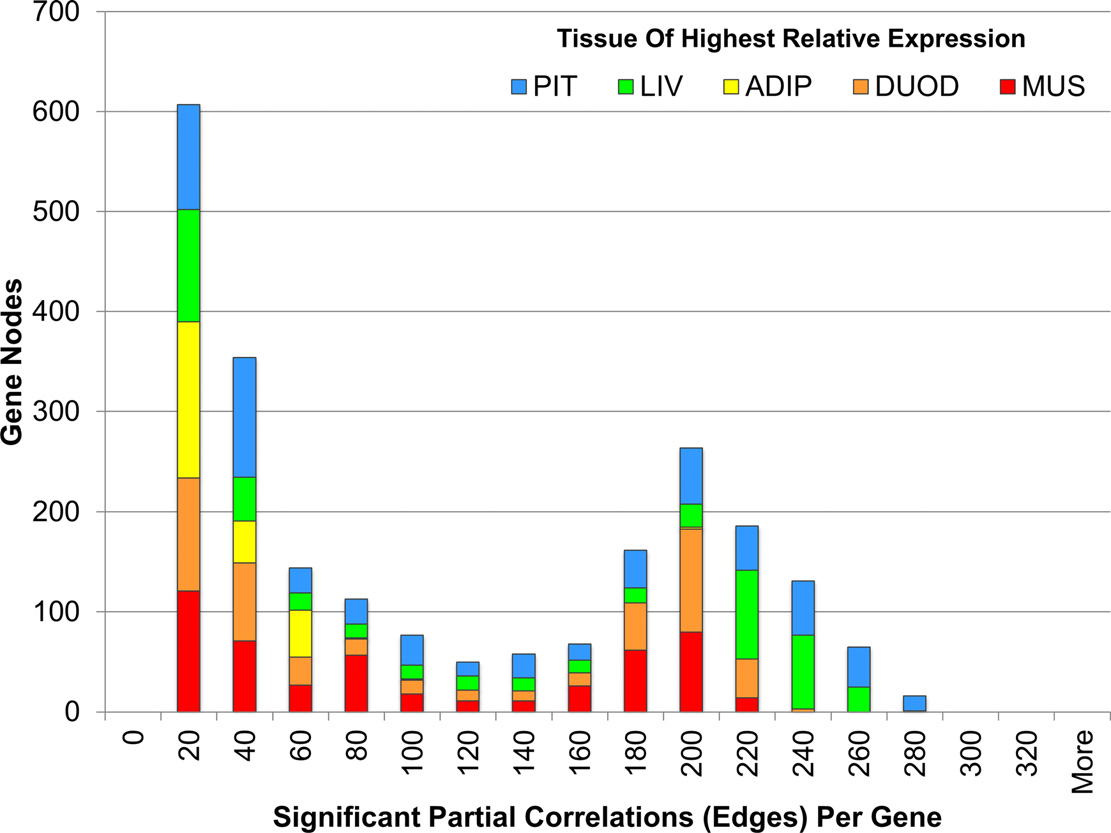
Distribution of significant partial correlations per gene in the co-expression network. This figure shows the number of significant partial correlations (or edges in the gene co-expression network) with other genes, ranging up to 280 per gene node. For each bin, genes are grouped by the tissue of highest relative expression, including pituitary (light blue), skeletal muscle (red), liver (light green), visceral adipose (yellow), and duodenum (orange), to give an approximation of the distribution within tissue. The pituitary, liver and duodenum are most highly represented among the genes with the highest number of edges.

There were 6 major clusters to the PCIT network, which align with the tissue of highest relative expression (with muscle having two clusters and all other tissues having one cluster per tissue), as more than 90% of edges are between genes within tissue. The number of edges from one tissue to another (e.g. from liver to any other tissue) were proportional to the number of nodes for that tissue (R^2^ = 0.94). Commensurate with its role as a hormonal regulator, of the top 50 genes ranked by number of edges to other genes, 38 are most highly expressed or tissue specific to the pituitary, such as luteinizing hormone/lutropin subunit beta (*LHB;* 280 edges), G protein-coupled receptor 173 (*GPR173*; 273 edges), EF-hand domain C-terminal containing 1 (*EFHC1*; 268 edges), prolactin (*PRL*; 267 edges), and growth hormone/somatotropin (*GH1*; 266 edges). The distribution of number of edges per gene is provided in Fig 4.

**Fig 5:**
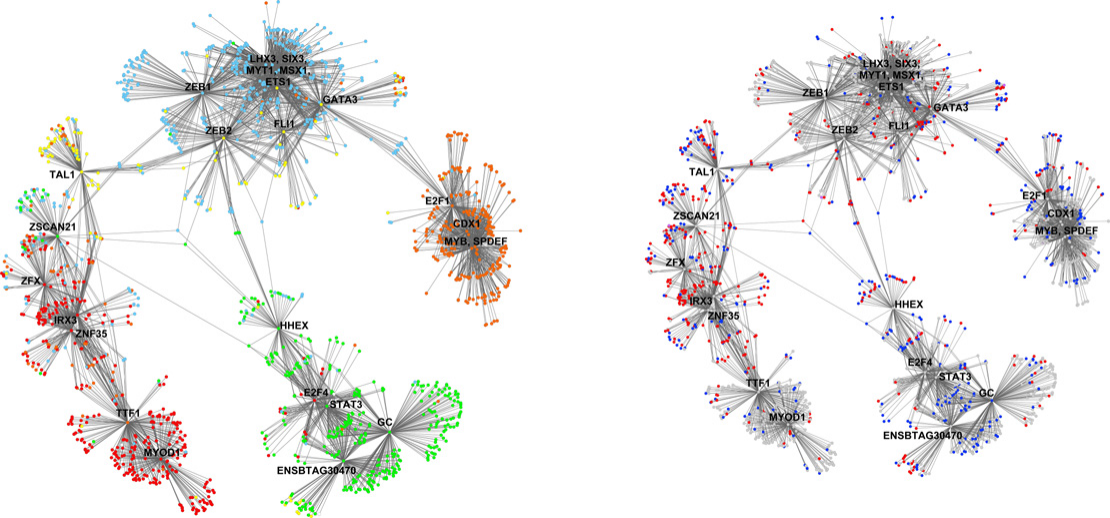
a and b. Gene co-expression network subset including all genes which are or are correlated with a transcription factor (N = 1,673). Genes (or nodes) are connected by an edge only when a significant partial correlation with a TF was found. Fig 5a displays nodes colored by tissue of highest relative expression for each gene: pituitary (light blue), skeletal muscle (red), liver (light green), visceral adipose (yellow), and duodenum (orange). The key transcription factor for each cluster is labeled. Fig 5b highlights differentially expressed genes (dark blue) and genes harboring GWAS SNP for RFI (red) or both (purple), with all other nodes colored grey.

The majority of edges are between TS genes (54%) or between TS and DE, TF, or SNP genes (32%). There are proportionally more edges to DE genes than to SNP genes (27% versus 20%). Two genes identified in Bolormaa et al. [19] as potential QTL for RFI were Src homology 2 domain containing transforming protein 3 (*SHC3*) and insulin-like growth factor binding protein 2 (*IGFBP2*). *SHC3*, while not DE or a TF, was significantly correlated with 18 DE genes (predominantly expressed in the pituitary and duodenum), 17 SNP genes, 5 TF [Msh homeobox 1 (*MSX1*), paired box 8 (*PAX8*), zinc finger protein 300 (*ZNF300*), v-ets avian erythroblastosis virus E26 oncogene homolog 1 (*ETS1*), and signal transducer and activator of transcription 6, interleukin-4 induced (*STAT6*)], and 1 TS gene. *IGFBP2* was adjacent to but did not harbor GWAS SNP, was not used as a SNP gene in this study nor was it identified as DE or TS, but related proteins *IGFBP1* and 4 were TS and DE and correlated with 219 and 18 genes in the liver, respectively. Five percent of edges connect to TF, of which the most highly connected TF are *MSX1* (235 edges), *GC* (228), v-myb avian myeloblastosis viral oncogene homolog (*MYB;* 203), *ETS1* (200), SIX homeobox 3 (*SIX3*; 194), SAM pointed domain containing ETS transcription factor (*SPDEF;* 192), zinc finger E-box-binding homeobox 1 (*ZEB1*; 187), LIM homeobox 3 (*LHX3*; 182), and myogenic differentiation 1 (*MYOD1*; 165). TF and genes connected to them by an edge were extracted and visualized as a new network (Fig 5).

In the TF network diagram, the clusters by tissue are present, with small clusters around TF that regulate gene expression within and across tissues. The TF with the greatest number of edges in the pituitary are, in descending order, *MSX1*, *ETS1*, *SIX3*, *ZEB1*, *LHX3*, myelin transcription factor 1 (*MYT1*), zinc finger E-box binding homeobox 2 (*ZEB2*), friend leukemia integration 1 transcription factor (*FLI1*), and GATA binding protein 3 (*GATA3*). *GATA3* is linked through DE and SNP genes to E2F transcription factor 1 (*E2F1*) in the duodenum, in which the other major TF are *MYB*, *SPDEF*, and caudal type homeobox 1 (*CDX1*). Similarly, *ZEB1* and *FLI1* are linked with hematopoietically expressed homeobox (*HHEX*) in the liver, in which *GC*, uncharacterized protein *ENSBTAG00000030470*, signal transducer and activator of transcription 3 (*STAT3*), and E2F transcription factor 4 (*E2F4)* are other major TF. *ZEB2* is linked with T-cell acute lymphocytic leukemia 1 (*TAL1)*, the only major adipose TF, which clusters with muscle TFs *MYOD1*, transcription termination factor, RNA polymerase I (*TTF1*), Zinc finger X-chromosomal protein (*ZFX*), iroquois homeobox 3 (*IRX3*), zinc finger and SCAN domain containing 21 (*ZSCAN21*), and zinc finger protein 35 (*ZNF35)*. Most clusters are an approximately even mixture of DE and SNP genes, with the exception of the *GC/ZNF160*-centric cluster in the liver which is richer in DE genes, and a cluster of SNP genes linked to *ZFX*, *IRX3*, and zinc finger protein 35 (*ZNF35*) in skeletal muscle. Comparing these SNP genes to the other muscle-specific gene clusters (centered around *MYOD1*, *TTF1*, and *ZSCAN21*), the *ZFX-IRX3-ZNF35* cluster displays enrichment of transcription regulation; nervous, connective, and blood vessel tissue development; metabolic activity; and immune response (Table 4).

**Table 4:** The top 10 enriched GO terms for the muscle specific gene clusters. Clusters are centered by the transcription factors *MYOD1TTF1*-*ZSCAN21* and *ZFX-IRX3*-*ZNF35*. The latter set of clusters had a higher proportion of SNP genes, suggesting conserved function in regulating RFI in beef cattle populations.

Enriched GO terms			
*MYOD1-TTF1-ZSCAN21* clusters		*ZFX-IRX3-ZNF35* clusters	
GO Term	P-value	GO Term	P-value
titin binding	8.39E-10	RNA polymerase II transcription factor binding, negative regulation of transcription	1.93E-04
sarcomere organization	3.63E-07	negative regulation of astrocyte differentiation	6.61E-04
troponin I binding	5.59E-06	sympathetic nervous system development	2.46E-03
tropomyosin binding	1.03E-05	lung connective tissue development	3.90E-03
skeletal muscle cell differentiation	4.73E-05	blood vessel endothelial cell migration involved in intussusceptive angiogenesis	3.90E-03
ventricular cardiac muscle tissue morphogenesis	4.91E-05	N-acetylglucosamine kinase activity	3.90E-03
transition between fast and slow fiber	7.57E-05	helper T cell enhancement of adaptive immune response	3.90E-03
ion channel binding	7.57E-05	cellular urea homeostasis	3.90E-03
calcium ion binding	1.44E-04	cellular creatinine homeostasis	3.90E-03
cardiac myofibril assembly	2.88E-04	cellular ammonia homeostasis	3.90E-03

## Discussion

This paper discussed the results of a progeny test of two Angus bulls with either high or low RFI genomic breeding value on the progeny for a variety of phenotypic traits collected from weaning until slaughter, multiple tissue transcriptomics, and gene co-expression network analysis. While conducted in a small population of Angus animals, it was found that the sire breeding value for RFI did predict differences in progeny phenotype for finishing period RFI. This is consistent with the widely observed accuracy and utility of genomic predictions in various beef and dairy cattle [6, 8, 26, 27]. These two bull sires differ in breeding value for many body weight traits (S1 Table), including mature and hot carcass weights, suggesting an increased growth rate among the progeny of the low RFI sire relative to those of the high RFI sire. Comparing predicted and observed ADG and DMI as well as RFI, a trend of increased ADG was observed in the finishing period with no difference in intake, in contrast to the predicted difference in genomic breeding values for DMI. This may be partially accounted for by the low n of our study and that RFI has been shown to be only lowly to moderately repeatable (0.29 to 0.62) [28–30]; however another cause could be differing trait definitions between the reference data (predominantly purebred bulls) and the test data (feedlot steers). American Angus DMI records depend on bull test data, which is generally collected before the bull is one year of age, making the DMI breeding value potentially more similar to a growing period DMI, which in our data showed a trend of decreased DMI in progeny of the low RFI sire relative to the high RFI sire. While the progeny groups show similar group differences in RFI in the growing and finishing period, phenotypic differences between them, including rate of gain and intake, change over time.

In the growing period, we observed a reduced number of feed bunk visits in progeny of the low RFI sire, which suggest that RFI is positively correlated with the number of daily feeding events, as has been observed in heifers and bulls in several beef and dairy breeds [31–33]. Feeding behavior was not recorded in the finishing phase, with the exception of the five days in which animals were fed from the GreenFeed methane emissions measurement unit, which based on the amount of variability observed, is too short a period to accurately gauge feeding behavior. We did not observe any differences in finishing period methane emissions, though this is not surprising given that methane production is highly correlated with DMI [34] and there was no difference in DMI between sire groups during this period. For future studies, it would be useful to measure both feeding events and methane emissions over a longer time interval, in order to increase accuracy and allow direct correlation with each other and DMI.

In the RNA-seq portion of this study, we found 633 differentially expressed genes between sire groups, of which one-fifth overlapped with the 1,026 tissue specific genes. Compared to a study by Canovas et al. [16] which used similar methods to study the effects of puberty in composite beef cattle, we found a comparable number of TS genes, but fewer DE and TF genes, indicating that genetic predisposition for feed efficiency are associated with more subtle changes in a variety of processes versus the massive cascade of change caused by puberty in developing heifers. While a variety of biofunctions were associated with the DE genes, reduced fat accumulation in adipose tissue in conjunction with an upregulation of muscle growth would agree with the reduced lean:fat ratio observed in the low RFI sire group carcasses based on carcass specific gravity. Similar effects have been observed from the application of growth promotants which increase growth through an increased lean muscle growth at the expense of fat deposition [35]. This effect is also seen less dramatically in cattle of larger mature size or which are slaughtered at an earlier stage of maturity [35]. In this experiment, low RFI sire progeny were both more feed efficient and possessing heavier body weights (as predicted from sire breeding values), contributing to an increased carcass protein:fat ratio.

Pathway analysis of the differentially expressed genes between progeny groups predicted an increased immune system and inflammatory response in the duodenum and other tissues. Increased liver inflammation and periportal lesions and altered lipid metabolism were also observed in more feed efficient Nellore beef bulls [36]. The increased inflammatory response in the progeny of the low RFI sire suggests perhaps a heightened acidosis or sensitivity to the high grain finishing diet while supporting an increased growth rate or body size. High concentrate feeding has been affect numerous inflammatory biomarkers such as increasing peak concentrations of plasma acute phase proteins [37], rumen lipopolysaccharide concentration, and serum amyloid-A and haptoglobin concentrations [38] relative to higher roughage diets. However, Paradis et al. [39] posited that more efficient beef heifers respond less to hepatic proinflammatory stimuli based on differential expression of interferon-modulated genes in the liver, and reduced inflammatory markers and increased alkaline phosphatase (which aids in detoxification) were also observed in low RFI swine [40]. These may indicate differential effects of diet, as developing heifers and swine are managed differently than finishing beef steers, or these may be effects that vary from population to population.

Examining the transcription factor sub-network, many of the key transcription factor hubs have been linked with feed efficiency, growth, intake, or carcass traits across species. Genes linked directly to feed efficiency include *IRX3* in Australian Angus cattle [41], *LHX3* in crossbred U.S. beef cattle [42], *MYOD1* in Landrace swine [43], and *ETS1* in Duroc swine [44] and commercial broiler chickens [45]. Other genes have been linked to muscle hypertrophy and lean growth, which was observed in this study to be enhanced in progeny of the low RFI sire. Polymorphisms in *MYOD1* have been associated with carcass and muscle traits in cattle [46] and swine [47]. *TTF1* and *APOA2*, a differentially expressed transcription factor in this study, have been associated with meat quality in Nelore cattle [48]. *SIX3* was found to be differentially expressed in double-muscled cattle [49]. In addition to direct effects on insulin secreting enzymes such as *PCSK2*, the transcription factors *HHEX* and *CDKNA2A/B* have been associated with impaired insulin release in diabetic humans [50]. In addition to direct observations of feeding behavior, some transcription factors such as *GATA3* and *STAT3* have been associated with eating behavior and appetite in mice and swine [51, 52], as both are linked to leptin-mediated control of satiety and inflammation [53, 54]. Further, *MYT1* regulates neuropeptide Y (*NPY*) expression [55], which stimulates feed intake and energy homeostasis [56]. *E2F1* was observed to be downregulated in the liver of swine expressing *MC4R* D298N variant, which is associated with increased feed intake, growth, and backfat thickness [57]. Lastly, Bolormaa et al. [58] suggested that *ZEB1* as a candidate for a QTL with pleiotropic effects on stature, fatness and reproduction in beef cattle. This study presents some evidence for linking these transcription factors, independently identified as impacting RFI and related traits, into a network associated with RFI in cattle possessing this set of ancillary phenotypes. Additional research should be performed to determine how consistently similar phenotypic trends are observed in more feed efficient beef cattle.

While supportive evidence has been outlined for some of the associations in this analysis, the overlap between the GWAS SNP genes and the DE genes was not high, although a large proportion could be linked to each other by partial correlations. For example, SNP genes *SHC3* and the *IGFBP* gene family possessed nearly 300 significant partial correlations to DE and TF genes, suggesting a potential regulatory network. The TF hubs in muscle also do not show an even distribution of DE and SNP genes, with different functions associated with DE-rich versus SNP-rich gene sub-networks. The simplest explanation is that many of the DE effects may be unique to this population and to the inherent differences between the two siring bulls. Another highly speculative possibility could be that the major TF affecting feed efficiency can manifest in DE differences along many different pathways, based on the specific populations, diets, and environments involved, as has been suggested in the context of complex human disease [59]. The results from this analysis, linking genomic prediction through transcriptomics and deep phenotyping, highlight some key connections but also the remaining questions surrounding the complex trait of RFI, pointing toward the highly polygenic nature of this trait.

This study provides a set of phenotypes across age and growth period identified as associated with genomically predicted differences in RFI between two Angus bull sires. Further, we identified differentially expressed genes and gene co-expression networks linking DE genes with tissue function, transcription factors, and genes harboring GWAS SNP. The information about significant genes and gene associations may be used as prior information of QTL for genomic prediction, in addition to its utility at defining pathways and regulatory networks. Due to the small size of this population, it is advised to validate associations through additional studies.

## Supporting Information

S1 Table Difference in sire breeding values for growth, intake, carcass, and reproductive traits. (DOCX)

S2 Table Position, mean expression, and fold change for differentially expressed genes (DE), tissue specific genes (TS), transcription factors (TF), and genes harboring GWAS SNP (SNP). Genes are ordered by tissue of highest expression and mean expression in that tissue.
(XLSX)
