# Supplementary material for: Identification of Gene Networks for Residual Feed Intake in Angus Cattle Using Genomic Prediction and RNA-seq
Source: PLoS One. 2016 Mar 28;11(3):e0152274. doi: 10.1371/journal.pone.0152274 (PMC4809598; doi:10.1371/journal.pone.0152274)
Supplement: S1 Table — (DOCX) [file pone.0152274.s001.docx]

**S1 Table. Difference in sire breeding values for growth, intake, carcass, and reproductive traits.**

| Trait | Difference in sire breeding values  (low RFI sire – high RFI sire) |
| --- | --- |
| Average daily gain, kg/d ^a^ | 0.00 |
| Dry matter intake, kg/d ^a^ | -0.35 |
| Residual feed intake, kg/d ^a^ | -0.32 |
| Birth weight, kg ^b^ | 4.45 |
| Weaning weight, kg ^b^ | 13.64 |
| Yearling weight, kg ^b^ | 24.55 |
| Yearling height, cm ^b^ | 3.56 |
| Residual average daily gain, kg/d ^b^ | 0.08 |
| Mature weight, kg ^b^ | 67.27 |
| Carcass weight, kg ^b^ | 20.91 |
| Rib-eye area, cm^2^ ^b^ | 11.48 |
| Marbling score ^b^ | 0.04 |
| Backfat thickness, mm ^b^ | -1.02 |
| Scrotal circumference, cm ^b^ | -2.68 |
| Heifer pregnancy rate, % ^b^ | 7.80 |

^a^From Zoetis genomic predictions, 2010 calibration

^b^From American Angus Association breeding values (2011)
